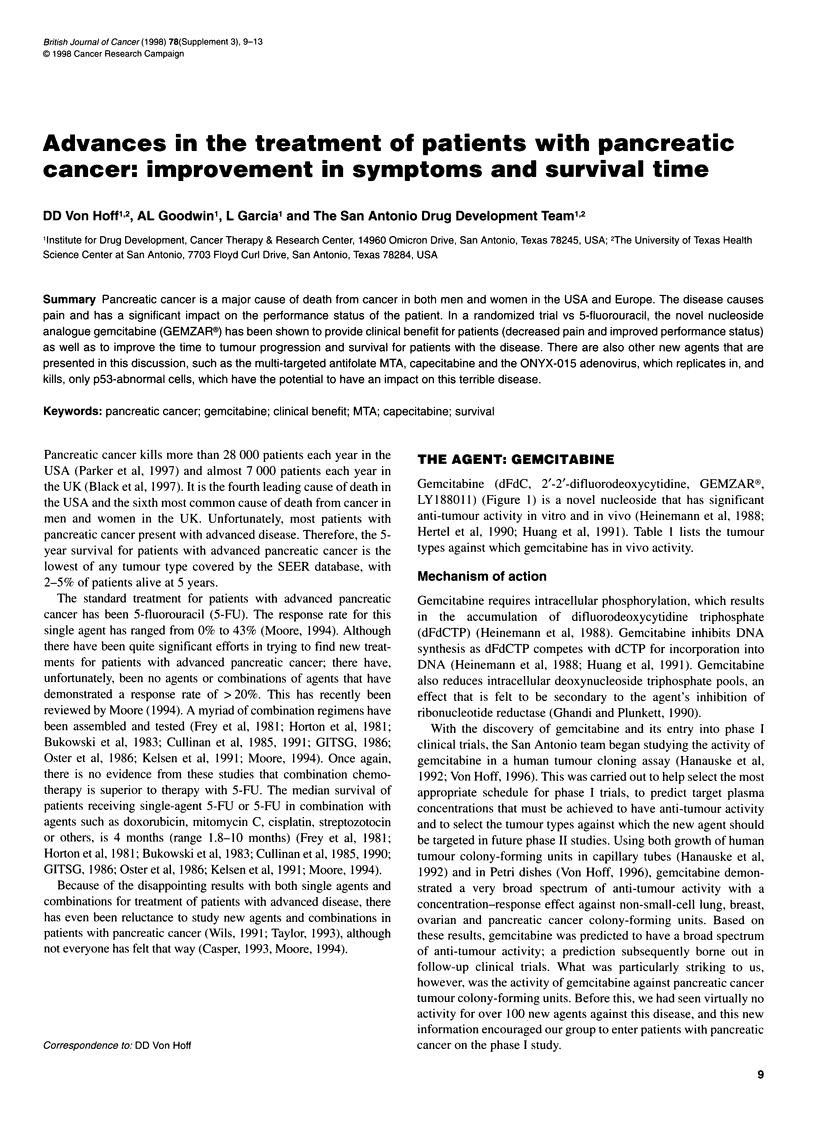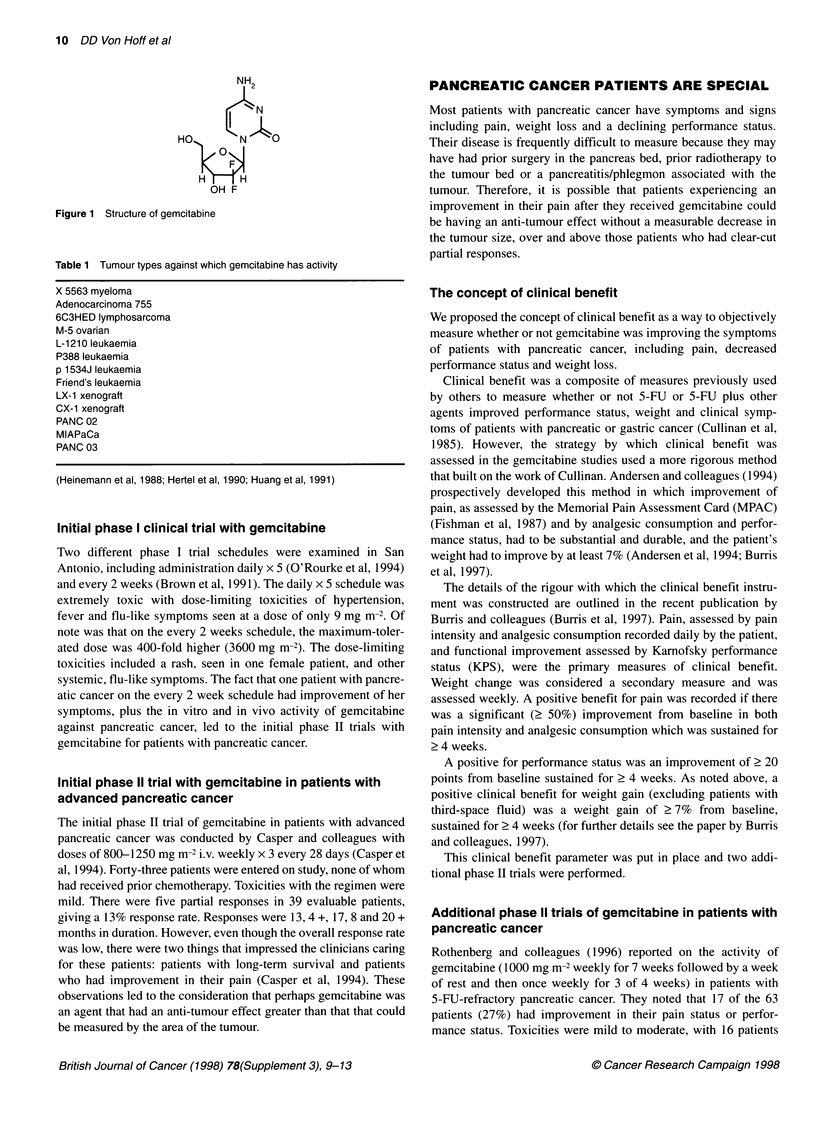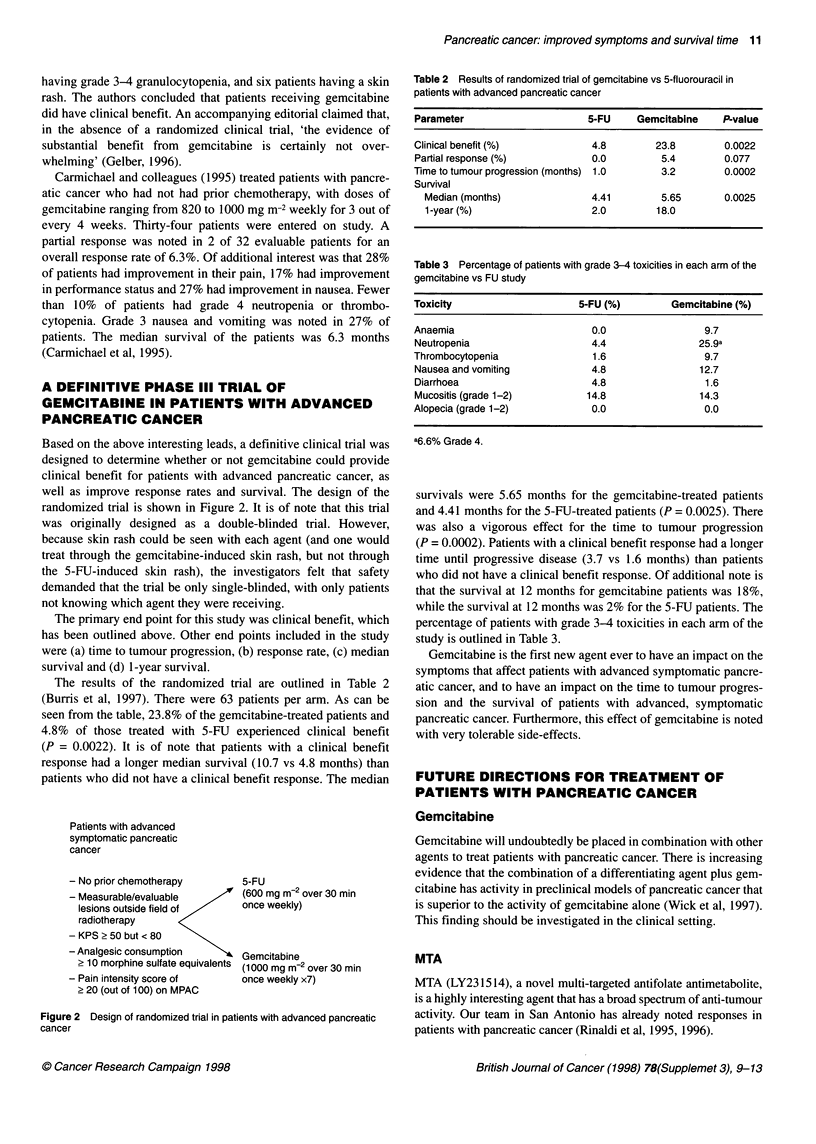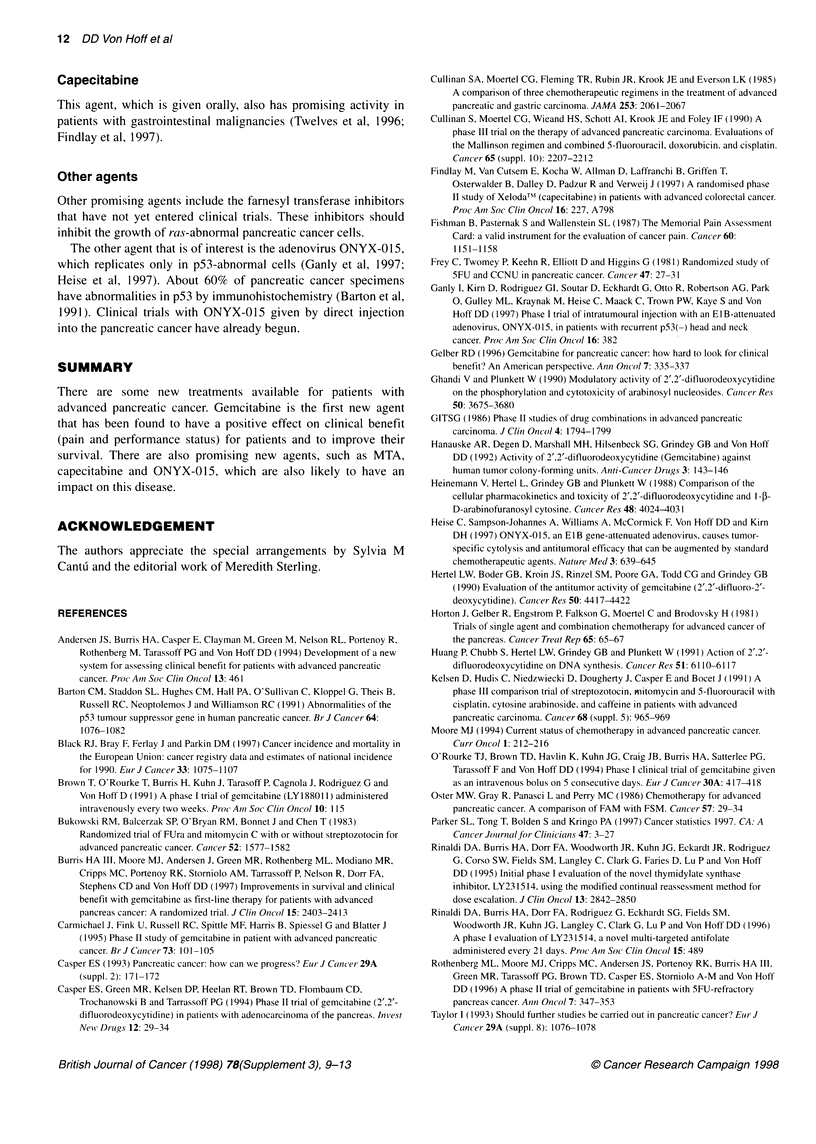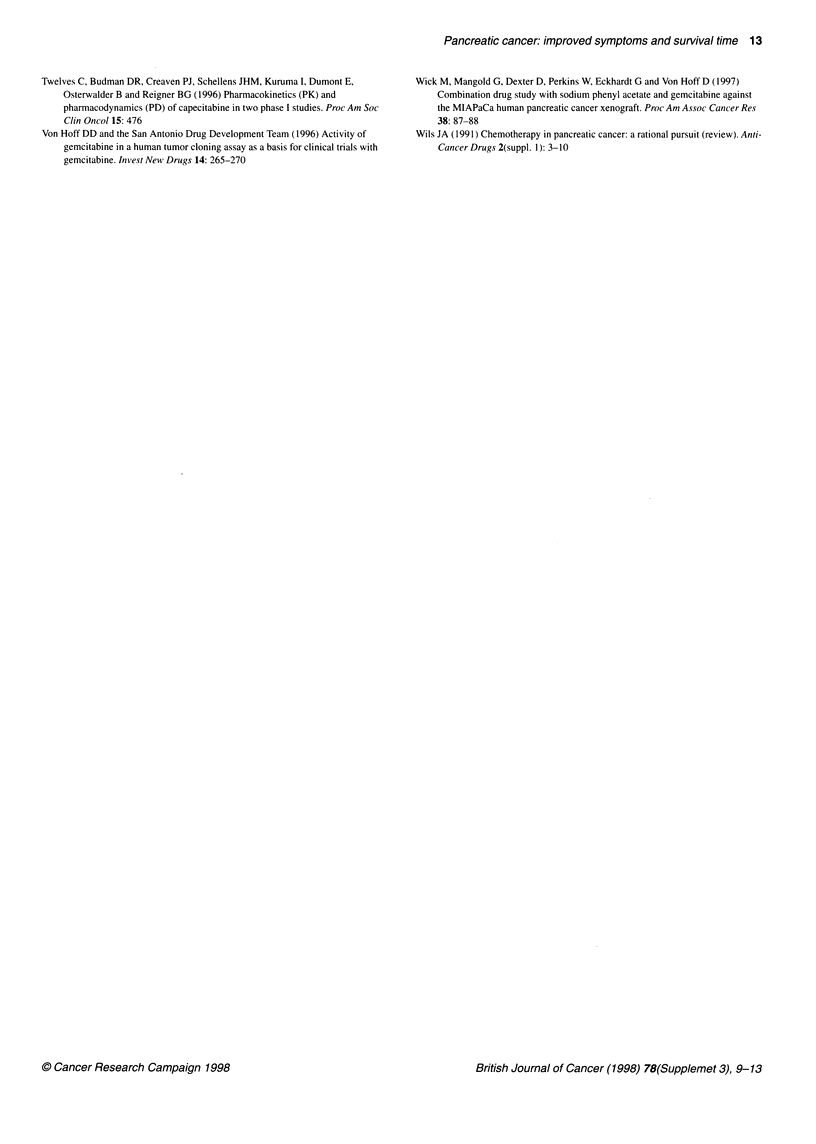# Advances in the treatment of patients with pancreatic cancer: improvement in symptoms and survival time. The San Antonio Drug Development Team.

**DOI:** 10.1038/bjc.1998.748

**Published:** 1998

**Authors:** D. D. Von Hoff, A. L. Goodwin, L. Garcia

**Affiliations:** Institute for Drug Development, Cancer Therapy & Research Center, San Antonio, Texas 78245, USA.

## Abstract

Pancreatic cancer is a major cause of death from cancer in both men and women in the USA and Europe. The disease causes pain and has a significant impact on the performance status of the patient. In a randomized trial vs 5-fluorouracil, the novel nucleoside analogue gemcitabine (GEMZAR) has been shown to provide clinical benefit for patients (decreased pain and improved performance status) as well as to improve the time to tumour progression and survival for patients with the disease. There are also other new agents that are presented in this discussion, such as the multi-targeted antifolate MTA, capecitabine and the ONYX-015 adenovirus, which replicates in, and kills, only p53-abnormal cells, which have the potential to have an impact on this terrible disease.


British Journal of Cancer (1998) 78(Supplement 3), 9–13
© 1998 Cancer Research Campaign

### Advances in the treatment of patients with pancreatic cancer: improvement in symptoms and survival time

**DD Von Hoff[1,2], AL Goodwin[1], L Garcia[1] and The San Antonio Drug Development Team[1,2]**

[1]Institute for Drug Development, Cancer Therapy & Research Center, 14960 Omicron Drive, San Antonio, Texas 78245, USA; [2]The University of Texas Health Science Center at San Antonio, 7703 Floyd Curl Drive, San Antonio, Texas 78284, USA

**Summary** Pancreatic cancer is a major cause of death from cancer in both men and women in the USA and Europe. The disease causes pain and has a significant impact on the performance status of the patient. In a randomized trial vs 5-fluorouracil, the novel nucleoside analogue gemcitabine (GEMZAR®) has been shown to provide clinical benefit for patients (decreased pain and improved performance status) as well as to improve the time to tumour progression and survival for patients with the disease. There are also other new agents that are presented in this discussion, such as the multi-targeted antifolate MTA, capecitabine and the ONYX-015 adenovirus, which replicates in, and kills, only p53-abnormal cells, which have the potential to have an impact on this terrible disease.

**Keywords:** pancreatic cancer; gemcitabine; clinical benefit; MTA; capecitabine; survival

Pancreatic cancer kills more than 28 000 patients each year in the USA (Parker et al, 1997) and almost 7 000 patients each year in the UK (Black et al, 1997). It is the fourth leading cause of death in the USA and the sixth most common cause of death from cancer in men and women in the UK. Unfortunately, most patients with pancreatic cancer present with advanced disease. Therefore, the 5-year survival for patients with advanced pancreatic cancer is the lowest of any tumour type covered by the SEER database, with 2–5% of patients alive at 5 years.

The standard treatment for patients with advanced pancreatic cancer has been 5-fluorouracil (5-FU). The response rate for this single agent has ranged from 0% to 43% (Moore, 1994). Although there have been quite significant efforts in trying to find new treatments for patients with advanced pancreatic cancer; there have, unfortunately, been no agents or combinations of agents that have demonstrated a response rate of > 20%. This has recently been reviewed by Moore (1994). A myriad of combination regimens have been assembled and tested (Frey et al, 1981; Horton et al, 1981; Bukowski et al, 1983; Cullinan et al, 1985, 1991; GITSG, 1986; Oster et al, 1986; Kelsen et al, 1991; Moore, 1994). Once again, there is no evidence from these studies that combination chemotherapy is superior to therapy with 5-FU. The median survival of patients receiving single-agent 5-FU or 5-FU in combination with agents such as doxorubicin, mitomycin C, cisplatin, streptozotocin or others, is 4 months (range 1.8–10 months) (Frey et al, 1981; Horton et al, 1981; Bukowski et al, 1983; Cullinan et al, 1985, 1990; GITSG, 1986; Oster et al, 1986; Kelsen et al, 1991; Moore, 1994).

Because of the disappointing results with both single agents and combinations for treatment of patients with advanced disease, there has even been reluctance to study new agents and combinations in patients with pancreatic cancer (Wils, 1991; Taylor, 1993), although not everyone has felt that way (Casper, 1993, Moore, 1994).

*Correspondence to:* DD Von Hoff

### THE AGENT: GEMCITABINE

Gemcitabine (dFdC, 2′-2′-difluorodeoxycytidine, GEMZAR®, LY188011) (Figure 1) is a novel nucleoside that has significant anti-tumour activity in vitro and in vivo (Heinemann et al, 1988; Hertel et al, 1990; Huang et al, 1991). Table 1 lists the tumour types against which gemcitabine has in vivo activity.

### Mechanism of action

Gemcitabine requires intracellular phosphorylation, which results in the accumulation of difluorodeoxycytidine triphosphate (dFdCTP) (Heinemann et al, 1988). Gemcitabine inhibits DNA synthesis as dFdCTP competes with dCTP for incorporation into DNA (Heinemann et al, 1988; Huang et al, 1991). Gemcitabine also reduces intracellular deoxynucleoside triphosphate pools, an effect that is felt to be secondary to the agent's inhibition of ribonucleotide reductase (Ghandi and Plunkett, 1990).

With the discovery of gemcitabine and its entry into phase I clinical trials, the San Antonio team began studying the activity of gemcitabine in a human tumour cloning assay (Hanauske et al, 1992; Von Hoff, 1996). This was carried out to help select the most appropriate schedule for phase I trials, to predict target plasma concentrations that must be achieved to have anti-tumour activity and to select the tumour types against which the new agent should be targeted in future phase II studies. Using both growth of human tumour colony-forming units in capillary tubes (Hanauske et al, 1992) and in Petri dishes (Von Hoff, 1996), gemcitabine demonstrated a very broad spectrum of anti-tumour activity with a concentration–response effect against non-small-cell lung, breast, ovarian and pancreatic cancer colony-forming units. Based on these results, gemcitabine was predicted to have a broad spectrum of anti-tumour activity; a prediction subsequently borne out in follow-up clinical trials. What was particularly striking to us, however, was the activity of gemcitabine against pancreatic cancer tumour colony-forming units. Before this, we had seen virtually no activity for over 100 new agents against this disease, and this new information encouraged our group to enter patients with pancreatic cancer on the phase I study.

**Figure 1** Structure of gemcitabine

**Table 1** Tumour types against which gemcitabine has activity

| |
|---|
| X 5563 myeloma |
| Adenocarcinoma 755 |
| 6C3HED lymphosarcoma |
| M-5 ovarian |
| L-1210 leukaemia |
| P388 leukaemia |
| p 1534J leukaemia |
| Friend's leukaemia |
| LX-1 xenograft |
| CX-1 xenograft |
| PANC 02 |
| MIAPaCa |
| PANC 03 |

(Heinemann et al, 1988; Hertel et al, 1990; Huang et al, 1991)

### Initial phase I clinical trial with gemcitabine

Two different phase I trial schedules were examined in San Antonio, including administration daily × 5 (O'Rourke et al, 1994) and every 2 weeks (Brown et al, 1991). The daily × 5 schedule was extremely toxic with dose-limiting toxicities of hypertension, fever and flu-like symptoms seen at a dose of only 9 mg $m^{-2}$. Of note was that on the every 2 weeks schedule, the maximum-tolerated dose was 400-fold higher (3600 mg $m^{-2}$). The dose-limiting toxicities included a rash, seen in one female patient, and other systemic, flu-like symptoms. The fact that one patient with pancreatic cancer on the every 2 week schedule had improvement of her symptoms, plus the in vitro and in vivo activity of gemcitabine against pancreatic cancer, led to the initial phase II trials with gemcitabine for patients with pancreatic cancer.

### Initial phase II trial with gemcitabine in patients with advanced pancreatic cancer

The initial phase II trial of gemcitabine in patients with advanced pancreatic cancer was conducted by Casper and colleagues with doses of 800–1250 mg $m^{-2}$ i.v. weekly × 3 every 28 days (Casper et al, 1994). Forty-three patients were entered on study, none of whom had received prior chemotherapy. Toxicities with the regimen were mild. There were five partial responses in 39 evaluable patients, giving a 13% response rate. Responses were 13, 4 +, 17, 8 and 20 + months in duration. However, even though the overall response rate was low, there were two things that impressed the clinicians caring for these patients: patients with long-term survival and patients who had improvement in their pain (Casper et al, 1994). These observations led to the consideration that perhaps gemcitabine was an agent that had an anti-tumour effect greater than that that could be measured by the area of the tumour.

### PANCREATIC CANCER PATIENTS ARE SPECIAL

Most patients with pancreatic cancer have symptoms and signs including pain, weight loss and a declining performance status. Their disease is frequently difficult to measure because they may have had prior surgery in the pancreas bed, prior radiotherapy to the tumour bed or a pancreatitis/phlegmon associated with the tumour. Therefore, it is possible that patients experiencing an improvement in their pain after they received gemcitabine could be having an anti-tumour effect without a measurable decrease in the tumour size, over and above those patients who had clear-cut partial responses.

### The concept of clinical benefit

We proposed the concept of clinical benefit as a way to objectively measure whether or not gemcitabine was improving the symptoms of patients with pancreatic cancer, including pain, decreased performance status and weight loss.

Clinical benefit was a composite of measures previously used by others to measure whether or not 5-FU or 5-FU plus other agents improved performance status, weight and clinical symptoms of patients with pancreatic or gastric cancer (Cullinan et al, 1985). However, the strategy by which clinical benefit was assessed in the gemcitabine studies used a more rigorous method that built on the work of Cullinan. Andersen and colleagues (1994) prospectively developed this method in which improvement of pain, as assessed by the Memorial Pain Assessment Card (MPAC) (Fishman et al, 1987) and by analgesic consumption and performance status, had to be substantial and durable, and the patient's weight had to improve by at least 7% (Andersen et al, 1994; Burris et al, 1997).

The details of the rigour with which the clinical benefit instrument was constructed are outlined in the recent publication by Burris and colleagues (Burris et al, 1997). Pain, assessed by pain intensity and analgesic consumption recorded daily by the patient, and functional improvement assessed by Karnofsky performance status (KPS), were the primary measures of clinical benefit. Weight change was considered a secondary measure and was assessed weekly. A positive benefit for pain was recorded if there was a significant (≥ 50%) improvement from baseline in both pain intensity and analgesic consumption which was sustained for ≥ 4 weeks.

A positive for performance status was an improvement of ≥ 20 points from baseline sustained for ≥ 4 weeks. As noted above, a positive clinical benefit for weight gain (excluding patients with third-space fluid) was a weight gain of ≥ 7% from baseline, sustained for ≥ 4 weeks (for further details see the paper by Burris and colleagues, 1997).

This clinical benefit parameter was put in place and two additional phase II trials were performed.

### Additional phase II trials of gemcitabine in patients with pancreatic cancer

Rothenberg and colleagues (1996) reported on the activity of gemcitabine (1000 mg $m^{-2}$ weekly for 7 weeks followed by a week of rest and then once weekly for 3 of 4 weeks) in patients with 5-FU-refractory pancreatic cancer. They noted that 17 of the 63 patients (27%) had improvement in their pain status or performance status. Toxicities were mild to moderate, with 16 patients

 © Cancer Research Campaign 1998

having grade 3–4 granulocytopenia, and six patients having a skin rash. The authors concluded that patients receiving gemcitabine did have clinical benefit. An accompanying editorial claimed that, in the absence of a randomized clinical trial, 'the evidence of substantial benefit from gemcitabine is certainly not overwhelming' (Gelber, 1996).

Carmichael and colleagues (1995) treated patients with pancreatic cancer who had not had prior chemotherapy, with doses of gemcitabine ranging from 820 to 1000 mg $m^{-2}$ weekly for 3 out of every 4 weeks. Thirty-four patients were entered on study. A partial response was noted in 2 of 32 evaluable patients for an overall response rate of 6.3%. Of additional interest was that 28% of patients had improvement in their pain, 17% had improvement in performance status and 27% had improvement in nausea. Fewer than 10% of patients had grade 4 neutropenia or thrombocytopenia. Grade 3 nausea and vomiting was noted in 27% of patients. The median survival of the patients was 6.3 months (Carmichael et al, 1995).

### A DEFINITIVE PHASE III TRIAL OF GEMCITABINE IN PATIENTS WITH ADVANCED PANCREATIC CANCER

Based on the above interesting leads, a definitive clinical trial was designed to determine whether or not gemcitabine could provide clinical benefit for patients with advanced pancreatic cancer, as well as improve response rates and survival. The design of the randomized trial is shown in Figure 2. It is of note that this trial was originally designed as a double-blinded trial. However, because skin rash could be seen with each agent (and one would treat through the gemcitabine-induced skin rash, but not through the 5-FU-induced skin rash), the investigators felt that safety demanded that the trial be only single-blinded, with only patients not knowing which agent they were receiving.

The primary end point for this study was clinical benefit, which has been outlined above. Other end points included in the study were (a) time to tumour progression, (b) response rate, (c) median survival and (d) 1-year survival.

The results of the randomized trial are outlined in Table 2 (Burris et al, 1997). There were 63 patients per arm. As can be seen from the table, 23.8% of the gemcitabine-treated patients and 4.8% of those treated with 5-FU experienced clinical benefit ($P$ = 0.0022). It is of note that patients with a clinical benefit response had a longer median survival (10.7 vs 4.8 months) than patients who did not have a clinical benefit response. The median

**Figure 2** Design of randomized trial in patients with advanced pancreatic cancer

**Table 2** Results of randomized trial of gemcitabine vs 5-fluorouracil in patients with advanced pancreatic cancer

| Parameter | 5-FU | Gemcitabine | *P*-value |
|---|---|---|---|
| Clinical benefit (%) | 4.8 | 23.8 | 0.0022 |
| Partial response (%) | 0.0 | 5.4 | 0.077 |
| Time to tumour progression (months) | 1.0 | 3.2 | 0.0002 |
| Survival | | | |
| Median (months) | 4.41 | 5.65 | 0.0025 |
| 1-year (%) | 2.0 | 18.0 | |

**Table 3** Percentage of patients with grade 3–4 toxicities in each arm of the gemcitabine vs FU study

| Toxicity | 5-FU (%) | Gemcitabine (%) |
|---|---|---|
| Anaemia | 0.0 | 9.7 |
| Neutropenia | 4.4 | 25.9[a] |
| Thrombocytopenia | 1.6 | 9.7 |
| Nausea and vomiting | 4.8 | 12.7 |
| Diarrhoea | 4.8 | 1.6 |
| Mucositis (grade 1–2) | 14.8 | 14.3 |
| Alopecia (grade 1–2) | 0.0 | 0.0 |

[a]6.6% Grade 4.

survivals were 5.65 months for the gemcitabine-treated patients and 4.41 months for the 5-FU-treated patients ($P$ = 0.0025). There was also a vigorous effect for the time to tumour progression ($P$ = 0.0002). Patients with a clinical benefit response had a longer time until progressive disease (3.7 vs 1.6 months) than patients who did not have a clinical benefit response. Of additional note is that the survival at 12 months for gemcitabine patients was 18%, while the survival at 12 months was 2% for the 5-FU patients. The percentage of patients with grade 3–4 toxicities in each arm of the study is outlined in Table 3.

Gemcitabine is the first new agent ever to have an impact on the symptoms that affect patients with advanced symptomatic pancreatic cancer, and to have an impact on the time to tumour progression and the survival of patients with advanced, symptomatic pancreatic cancer. Furthermore, this effect of gemcitabine is noted with very tolerable side-effects.

### FUTURE DIRECTIONS FOR TREATMENT OF PATIENTS WITH PANCREATIC CANCER

### Gemcitabine

Gemcitabine will undoubtedly be placed in combination with other agents to treat patients with pancreatic cancer. There is increasing evidence that the combination of a differentiating agent plus gemcitabine has activity in preclinical models of pancreatic cancer that is superior to the activity of gemcitabine alone (Wick et al, 1997). This finding should be investigated in the clinical setting.

### MTA

MTA (LY231514), a novel multi-targeted antifolate antimetabolite, is a highly interesting agent that has a broad spectrum of anti-tumour activity. Our team in San Antonio has already noted responses in patients with pancreatic cancer (Rinaldi et al, 1995, 1996).

© Cancer Research Campaign 1998 

### Capecitabine

This agent, which is given orally, also has promising activity in patients with gastrointestinal malignancies (Twelves et al, 1996; Findlay et al, 1997).

### Other agents

Other promising agents include the farnesyl transferase inhibitors that have not yet entered clinical trials. These inhibitors should inhibit the growth of *ras*-abnormal pancreatic cancer cells.

The other agent that is of interest is the adenovirus ONYX-015, which replicates only in p53-abnormal cells (Ganly et al, 1997; Heise et al, 1997). About 60% of pancreatic cancer specimens have abnormalities in p53 by immunohistochemistry (Barton et al, 1991). Clinical trials with ONYX-015 given by direct injection into the pancreatic cancer have already begun.

### SUMMARY

There are some new treatments available for patients with advanced pancreatic cancer. Gemcitabine is the first new agent that has been found to have a positive effect on clinical benefit (pain and performance status) for patients and to improve their survival. There are also promising new agents, such as MTA, capecitabine and ONYX-015, which are also likely to have an impact on this disease.

### ACKNOWLEDGEMENT

The authors appreciate the special arrangements by Sylvia M Cantú and the editorial work of Meredith Sterling.

### REFERENCES

Andersen JS, Burris HA, Casper E, Clayman M, Green M, Nelson RL, Portenoy R, Rothenberg M, Tarassoff PG and Von Hoff DD (1994) Development of a new system for assessing clinical benefit for patients with advanced pancreatic cancer. *Proc Am Soc Clin Oncol* **13**: 461

Barton CM, Staddon SL, Hughes CM, Hall PA, O'Sullivan C, Kloppel G, Theis B, Russell RC, Neoptolemos J and Williamson RC (1991) Abnormalities of the p53 tumour suppressor gene in human pancreatic cancer. *Br J Cancer* **64**: 1076–1082

Black RJ, Bray F, Ferlay J and Parkin DM (1997) Cancer incidence and mortality in the European Union: cancer registry data and estimates of national incidence for 1990. *Eur J Cancer* **33**: 1075–1107

Brown T, O'Rourke T, Burris H, Kuhn J, Tarasoff P, Cagnola J, Rodriguez G and Von Hoff D (1991) A phase I trial of gemcitabine (LY188011) administered intravenously every two weeks. *Proc Am Soc Clin Oncol* **10**: 115

Bukowski RM, Balcerzak SP, O'Bryan RM, Bonnet J and Chen T (1983) Randomized trial of FUra and mitomycin C with or without streptozotocin for advanced pancreatic cancer. *Cancer* **52**: 1577–1582

Burris HA III, Moore MJ, Andersen J, Green MR, Rothenberg ML, Modiano MR, Cripps MC, Portenoy RK, Storniolo AM, Tarrassoff P, Nelson R, Dorr FA, Stephens CD and Von Hoff DD (1997) Improvements in survival and clinical benefit with gemcitabine as first-line therapy for patients with advanced pancreas cancer: A randomized trial. *J Clin Oncol* **15**: 2403–2413

Carmichael J, Fink U, Russell RC, Spittle MF, Harris B, Spiessel G and Blatter J (1995) Phase II study of gemcitabine in patient with advanced pancreatic cancer. *Br J Cancer* **73**: 101–105

Casper ES (1993) Pancreatic cancer: how can we progress? *Eur J Cancer* **29A** (suppl. 2): 171–172

Casper ES, Green MR, Kelsen DP, Heelan RT, Brown TD, Flombaum CD, Trochanowski B and Tarrassoff PG (1994) Phase II trial of gemcitabine (2′,2′-difluorodeoxycytidine) in patients with adenocarcinoma of the pancreas. *Invest New Drugs* **12**: 29–34

Cullinan SA, Moertel CG, Fleming TR, Rubin JR, Krook JE and Everson LK (1985) A comparison of three chemotherapeutic regimens in the treatment of advanced pancreatic and gastric carcinoma. *JAMA* **253**: 2061–2067

Cullinan S, Moertel CG, Wieand HS, Schott AI, Krook JE and Foley IF (1990) A phase III trial on the therapy of advanced pancreatic carcinoma. Evaluations of the Mallinson regimen and combined 5-fluorouracil, doxorubicin, and cisplatin. *Cancer* **65** (suppl. 10): 2207–2212

Findlay M, Van Cutsem E, Kocha W, Allman D, Laffranchi B, Griffen T, Osterwalder B, Dalley D, Padzur R and Verweij J (1997) A randomised phase II study of Xeloda™ (capecitabine) in patients with advanced colorectal cancer. *Proc Am Soc Clin Oncol* **16**: 227, A798

Fishman B, Pasternak S and Wallenstein SL (1987) The Memorial Pain Assessment Card: a valid instrument for the evaluation of cancer pain. *Cancer* **60**: 1151–1158

Frey C, Twomey P, Keehn R, Elliott D and Higgins G (1981) Randomized study of 5FU and CCNU in pancreatic cancer. *Cancer* **47**: 27–31

Ganly I, Kirn D, Rodriguez GI, Soutar D, Eckhardt G, Otto R, Robertson AG, Park O, Gulley ML, Kraynak M, Heise C, Maack C, Trown PW, Kaye S and Von Hoff DD (1997) Phase I trial of intratumoural injection with an E1B-attenuated adenovirus, ONYX-015, in patients with recurrent p53(–) head and neck cancer. *Proc Am Soc Clin Oncol* **16**: 382

Gelber RD (1996) Gemcitabine for pancreatic cancer: how hard to look for clinical benefit? An American perspective. *Ann Oncol* **7**: 335–337

Ghandi V and Plunkett W (1990) Modulatory activity of 2′,2′-difluorodeoxycytidine on the phosphorylation and cytotoxicity of arabinosyl nucleosides. *Cancer Res* **50**: 3675–3680

GITSG (1986) Phase II studies of drug combinations in advanced pancreatic carcinoma. *J Clin Oncol* **4**: 1794–1799

Hanauske AR, Degen D, Marshall MH, Hilsenbeck SG, Grindey GB and Von Hoff DD (1992) Activity of 2′,2′-difluorodeoxycytidine (Gemcitabine) against human tumor colony-forming units. *Anti-Cancer Drugs* **3**: 143–146

Heinemann V, Hertel L, Grindey GB and Plunkett W (1988) Comparison of the cellular pharmacokinetics and toxicity of 2′,2′-difluorodeoxycytidine and 1-β-D-arabinofuranosyl cytosine. *Cancer Res* **48**: 4024–4031

Heise C, Sampson-Johannes A, Williams A, McCormick F, Von Hoff DD and Kirn DH (1997) ONYX-015, an E1B gene-attenuated adenovirus, causes tumor-specific cytolysis and antitumoral efficacy that can be augmented by standard chemotherapeutic agents. *Nature Med* **3**: 639–645

Hertel LW, Boder GB, Kroin JS, Rinzel SM, Poore GA, Todd CG and Grindey GB (1990) Evaluation of the antitumor activity of gemcitabine (2′,2′-difluoro-2′-deoxycytidine). *Cancer Res* **50**: 4417–4422

Horton J, Gelber R, Engstrom P, Falkson G, Moertel C and Brodovsky H (1981) Trials of single agent and combination chemotherapy for advanced cancer of the pancreas. *Cancer Treat Rep* **65**: 65–67

Huang P, Chubb S, Hertel LW, Grindey GB and Plunkett W (1991) Action of 2′,2′-difluorodeoxycytidine on DNA synthesis. *Cancer Res* **51**: 6110–6117

Kelsen D, Hudis C, Niedzwiecki D, Dougherty J, Casper E and Bocet J (1991) A phase III comparison trial of streptozotocin, mitomycin and 5-fluorouracil with cisplatin, cytosine arabinoside, and caffeine in patients with advanced pancreatic carcinoma. *Cancer* **68** (suppl. 5): 965–969

Moore MJ (1994) Current status of chemotherapy in advanced pancreatic cancer. *Curr Oncol* **1**: 212–216

O'Rourke TJ, Brown TD, Havlin K, Kuhn JG, Craig JB, Burris HA, Satterlee PG, Tarassoff F and Von Hoff DD (1994) Phase I clinical trial of gemcitabine given as an intravenous bolus on 5 consecutive days. *Eur J Cancer* **30A**: 417–418

Oster MW, Gray R, Panasci L and Perry MC (1986) Chemotherapy for advanced pancreatic cancer. A comparison of FAM with FSM. *Cancer* **57**: 29–34

Parker SL, Tong T, Bolden S and Kringo PA (1997) Cancer statistics 1997. *CA: A Cancer Journal for Clinicians* **47**: 3–27

Rinaldi DA, Burris HA, Dorr FA, Woodworth JR, Kuhn JG, Eckardt JR, Rodriguez G, Corso SW, Fields SM, Langley C, Clark G, Faries D, Lu P and Von Hoff DD (1995) Initial phase I evaluation of the novel thymidylate synthase inhibitor, LY231514, using the modified continual reassessment method for dose escalation. *J Clin Oncol* **13**: 2842–2850

Rinaldi DA, Burris HA, Dorr FA, Rodriguez G, Eckhardt SG, Fields SM, Woodworth JR, Kuhn JG, Langley C, Clark G, Lu P and Von Hoff DD (1996) A phase I evaluation of LY231514, a novel multi-targeted antifolate administered every 21 days. *Proc Am Soc Clin Oncol* **15**: 489

Rothenberg ML, Moore MJ, Cripps MC, Andersen JS, Portenoy RK, Burris HA III, Green MR, Tarassoff PG, Brown TD, Casper ES, Storniolo A-M and Von Hoff DD (1996) A phase II trial of gemcitabine in patients with 5FU-refractory pancreas cancer. *Ann Oncol* **7**: 347–353

Taylor I (1993) Should further studies be carried out in pancreatic cancer? *Eur J Cancer* **29A** (suppl. 8): 1076–1078

© Cancer Research Campaign 1998

Twelves C, Budman DR, Creaven PJ, Schellens JHM, Kuruma I, Dumont E, Osterwalder B and Reigner BG (1996) Pharmacokinetics (PK) and pharmacodynamics (PD) of capecitabine in two phase I studies. *Proc Am Soc Clin Oncol* **15**: 476

Von Hoff DD and the San Antonio Drug Development Team (1996) Activity of gemcitabine in a human tumor cloning assay as a basis for clinical trials with gemcitabine. *Invest New Drugs* **14**: 265–270

Wick M, Mangold G, Dexter D, Perkins W, Eckhardt G and Von Hoff D (1997) Combination drug study with sodium phenyl acetate and gemcitabine against the MIAPaCa human pancreatic cancer xenograft. *Proc Am Assoc Cancer Res* **38**: 87–88

Wils JA (1991) Chemotherapy in pancreatic cancer: a rational pursuit (review). *Anti-Cancer Drugs* **2**(suppl. 1): 3–10

© Cancer Research Campaign 1998